# Caution against precaution: A case report on silent hypoxia in COVID-19

**DOI:** 10.1016/j.amsu.2020.11.007

**Published:** 2020-11-04

**Authors:** Ali Lari, Mohammad Alherz, Abdullah Nouri, Lotfi Botras, Salah Taqi

**Affiliations:** Intensive Care Department, Jaber Al-Ahmad Hospital, Ministry of Health, Kuwait

**Keywords:** Silent hypoxia, COVID-19, Intubation, Intensive care

## Abstract

**Introduction:**

Silent hypoxia is an entity that has been described in patients diagnosed with COVID-19. It is typically described as objective hypoxia in the absence of proportional respiratory distress. The physiological basis for this phenomenon is controversial, and its prognostic value is unclear. We present a case below, of a 66-year-old female presenting with severe hypoxia that was managed without mechanical ventilation.

**Presentation of case:**

A 66 year old female with multiple comorbidities initially presented with a cough, fever and an oxygen saturation of 70% on room air in the absence of respiratory distress or altered mentation. She subsequently tested positive for COVID-19 and was admitted to the intensive care unit; received oxygen via high flow nasal cannula and continuous positive pressure mask. The patient remained in the intensive care unit for 40 days under close observation and exhibited multiple episodes of silent hypoxia on weaning oxygen. She was discharged on room air with an oxygen saturation >90% after 56 days. The patient was not intubated during her stay.

**Discussion and conclusion:**

Clinicians face a clinical dilemma on whether to intubate a “silently hypoxemic” patient, who displays hypoxia out of proportion to clinical examination. The decision is confounded by a lack of clear evidence on whether the benefits of precautionary intubation outweighs the risks, especially in the current COVID-19 pandemic. A recent paradigm shift that recommends delaying intubation further displays the need for clearer analysis of the situation. Our case demonstrates a favorable outcome of the latter approach, yet emphasizes a case-by-case approach until clearer recommendations are available.

## Highlights

• COVID-19 may cause severe hypoxia in the absence of respiratory distress.
• The recommendations for intubating silently hypoxemic patients are unclear.
• Pre-emptive intubation versus close observation is currently debatable.
• The benefit to risk ratio of intubation should be considered carefully when determining management.

## 1 Introduction

Silent hypoxia is an entity that describes objective hypoxia in the absence of signs and symptoms of respiratory distress that is proportionate to the findings. Hypoxia is typically in the form of decreased oxygen saturation (SpO2) on pulse oximetry and hypoxemic blood gases. Both of the aforementioned tests reveal levels that are normally incompatible with life [1,8]. In the current COVID-19 pandemic, reports of silent hypoxia have surfaced [3,9], inviting various descriptions on the physiology and prognostic value of the phenomenon [5]. However, the true consequences of silent hypoxia remain controversial, leading to unclear recommendations on how to approach such cases.

In the following case report, we describe a 66-year-old female patient with COVID-19 who displayed severe hypoxia in an apparent absence of a proportionate respiratory response. We highlight particular difficulties in managing such cases and discuss the controversy regarding pre-emptive intubation. Silent hypoxia has previously been regarded as a harbinger of impending clinical deterioration and poor prognosis. Our case however, exhibits a favorable outcome while managing the patient conservatively. This case was reported in line with the CARE criteria [10].

### 1.1 Case presentation

A 66 year old woman presented via ambulance to the emergency department with a 3-day history of fever, dry cough and fatigue, on a background of asthma, ischemic heart disease, congestive cardiac failure, obstructive sleep apnea using home continuous positive airway pressure, obesity, chronic kidney disease, hypothyroidism, diabetes mellitus and hypertension.

**Fig. 1:**
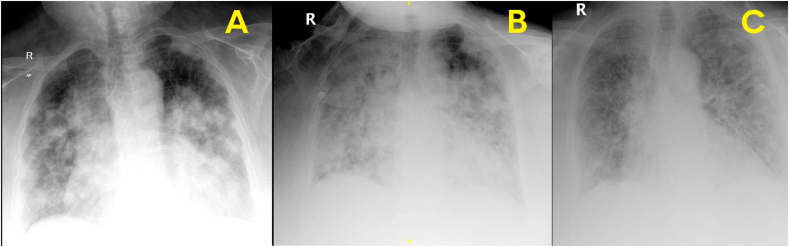
A) Admission Chest X-Ray; B) Day 10 Chest X-Ray; C) Discharge chest X-Ray.

Initial assessment showed a room-air oxygen saturation (SpO2) of 70%, in the absence of respiratory distress or altered mentation. Following admission and confirmation of SARS-CoV-2 infection, she was admitted to the COVID-19-dedicated intensive care unit (ICU) on the premise of persistent hypoxia. Arterial blood gases (ABG) revealed a PaO2 58 mmHg, PaCO2 46 mmHg, SpO2 86% while receiving 15 L/min via non-rebreather mask (NRBM). Severe bilateral infiltrates were seen on a chest radiograph (Fig. 1). However, the patient remained alert and oriented; the heart rate was 65 beats per minute and respiratory rate (RR) 25 breaths per minute regardless of SpO2 fluctuations. Accessory muscles of respiration were not being used and she denied any significant breathing difficulty. Treatment was escalated to high-flow nasal oxygen/cannula (HFNO/C); FiO2 100%, flow 40L/min, with an immediate improvement of SpO2 to >88%.

Laboratory investigations were repeated regularly, including inflammatory markers to guide steroid therapy. The patient received therapeutic anticoagulation, a 10-day course of dexamethasone, empirical antibiotic coverage, appropriate analgesia and intensive physiotherapy (Table .1). An arterial line was in place for regular blood gas sampling, along with a peripheral venous cannula and a urinary catheter. Prone positioning was not tolerated by the patient, and thus we aimed to avoid a supine position by keeping the patient seated <45° or in the left lateral position. HFNO was continuously supplied and the CPAP mask was only given overnight. Any significant oxygen desaturation was met with careful reassessment, an increase in oxygen, and a consideration of intubation as an option.

**Table 1:** Displaying medication received.

Medication	Dose	Frequency	Route	Duration (days)
Dexamethasone	6mg	Od	Oral	10
Enoxaparin	80mg	Bd	S/C	30
Piperacillin/tazobactam	2.5g	Tds	IV	7
Omeprazole	40mg	Od	IV	35
Paracetamol	1g	PRN	IV	35
Fentanyl	50 mcg/hr	Every 3 days	Patch	9

Abbreviation smg; milligrams, mcg; micrograms, Od; once daily, Bd; twice daily, Tds: three times daily,; PRN; as required.

Our monitoring strategy to assess for deterioration/improvement and the need for intubation consisted of parameters including; daily clinical examination, vital signs, blood gases and lactate. Particular attention was paid to the patient's mental status and reported symptoms such as exhaustion and dyspnea. We established intubation criteria that entail: significant dyspnea, RR > 35–40 at rest, exhaustion and requests to be intubated; severe hypoxemic or acidotic blood gases PaO2 <40 mmHg, inability to maintain SpO2 of 88% throughout the majority of the day at rest and altered mental status. We also considered signs of systemic hypoperfusion such as; progressive organ failure, hypotension and elevation of lactate >2mmol/L. In addition, previous baseline parameters including; blood gases, SpO2 and respiratory rate were unclear to us. Considering the medical comorbidities, we presumed that this patient was unlikely to achieve a normal PaO2 and SpO2 at baseline. In our scenario, considerable thought was given to predict the potential outcome and hazards of intubation. The medical comorbidities and physiological status of our patient were a source of concern, yet also served as a basis for our preference not to intubate.

**Fig. 2:**
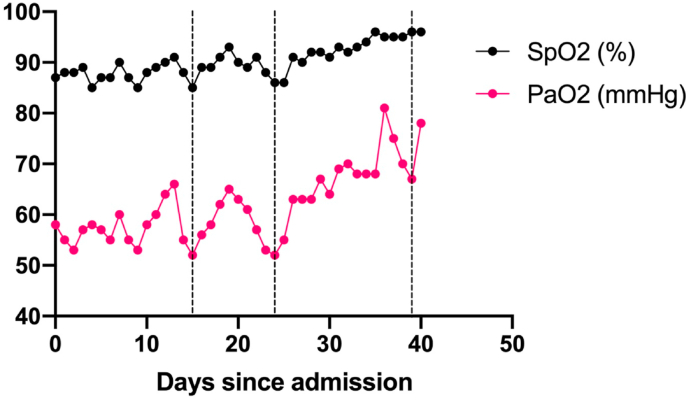
**Persistent episodes of silent hypoxemia following attempts to reduce oxygen supplementation.** A line graph of the fluctuations in oxygenation parameters since admission. Dotted lines denote attempts at oxygen supplementation reduction as determined by a fall in PaO2. No considerable changes to the patient's respiratory effort or general condition were observed. SpO2 (%) averages were calculated on a daily basis.

The patient remained in the ICU for 40 days. Throughout the course of her stay, weaning her oxygen supplementation proved a challenge, with our endeavors resulting in oxygen desaturation, in the absence of a considerable effect on respiratory effort (Fig. 2). She continued to receive oxygen via HFNC and simultaneously used overnight continuous positive airway pressure mask (CPAP) for 30 days.

On day 30 of her stay, our patient started improving and weaning was possible. The patient was tolerating NRBM 10–15L and maintaining a SpO2 >90% and ABG displayed a PaO2 of 80 mmHg and PaCO2 of 45 mmHg. The patient could comfortably tolerate oral feeding and carry a normal conversation. The patient was transferred to the ward on a NRBM 5L/min. The patient spent an uneventful 16 days in the ward on minimal oxygen therapy. She was subsequently weaned off oxygen supplementation and monitored to ensure SpO2 was maintained at rest and activity. The patient was finally discharged after a total hospital stay of 56 days. Despite the clear clinical improvement, treatment was prolonged in the ward, due to the lack of a clear consensus on how to manage such patients in the long term, and what the discharge criteria may entail. Ultimately, discharge criteria necessitated that firstly; SpO2 was >94% at rest, activities of daily living and exertion (e.g. walking for 5–10min), activities were tailored to patient's baseline. Next, the patient did not complain of significant symptoms dyspnea, fatigue, reduced oral intake and fever indicating a new-onset infection. Lastly, we ensured that laboratory parameters were acceptable (e.g. normal ABG, renal function test) and that radiological parameters were improving.

The patient was followed up at 4 weeks and 8 weeks post-discharge in the internal medicine and pulmonology outpatient department. A history, examination and basic vital signs were measured. She did not complain of dyspnea, fatigue and any residual symptoms. Oxygen saturation was 97% at rest. The patient was mobilizing well at home and was scheduled for follow up in 3 months’ time.

## 2 Discussion

A frequent yet challenging decision facing clinicians during the COVID-19 pandemic is whether to intubate a “silently-hypoxemic” patient. This refers to a patient who despite their poor oxygen levels not only appears to make no additional respiratory effort, but possesses normal faculties and their general state is incongruous with their numerical observations [1]. While the definition of silent hypoxia may vary, hypoxia out of proportion to the patient's symptoms is normally present. Debate is ongoing on whether this state is a predictor of a suddenly deteriorating picture and warrants liberal intubation [2,3], or whether a more careful deliberation combined with close observation is sufficient in light of the potential hazards of intervention [4]. The above case describes the course of the latter approach, yielding a favorable yet by no means guaranteed outcome.

This dilemma carries considerable ethical and clinical weight. On the one hand, the perceived oxygenation parameters are typically at least co-existent with some indication for intubation in various scenarios. As such, a prolonged wait may be perceived to contribute to avoidable morbidity and mortality, as well as the potential for hazardous emergency intubations. The latter may be further delayed by the logistical issues of contact with a COVID-19 positive patient; donning personal protective equipment affected by a time constraint.

On the other hand, opting to pre-emptively sedate and intubate a steadily breathing, talking patient without an end in sight given the relatively uncharted and prolonged course of the disease, is precarious to say the least. This is compounded by the inability to disentangle the evidence on whether intubation can actually improve outcomes, and the resultant lack of a definitive guideline. The early approach toward liberal intubation and ventilation [2] was later resisted by arguments supported to some extent by cases of successful conservative measures [4]. Further, the proposed physiological basis for the silent hypoxemia indicates that compensatory mechanisms may initially improve oxygen extraction via a shift in the dissociation curve, and fears of end-organ damage due to a low PaO2 are difficult to materialise unless a critical threshold is reached below 40 mmHg [5].

Particular considerations to make in light of the pandemic are the increased risk of nosocomial, drug-resistant and superimposed infections [6] coupled with the scarcity of equipment in some settings [7]. Additional general risks of mechanical ventilation include lung injury and severe infections requiring aggressive management with systemic antibiotics, sedatives, corticosteroids and vasopressors administered through central venous catheters. As such, while pre-emptive intubation may seem like the safe option in the face of silent hypoxemia, a decision not to intubate in this context may prove to yield the better outcomes. Until a clearer view emerges, decisions will be better served on a case-by-case basis and according to the setting of the treating facility.

This case highlights the controversy and difficulty in approaching silent hypoxia. The entity itself is variable in its outcomes, and management is equally challenging. The patient's complaints are essential in clinical decision-making and continuous close observation is required to uncover subtle signs of deterioration. Providers who encounter such presentations should also ensure to revise the latest literature on silent hypoxia.

## Provenance and peer review

Not commissioned, externally peer-reviewed.

## References

[1] Tobin M.J., Laghi F., Jubran A. (2020). ‘Why COVID-19 silent hypoxemia is baffling to physicians’, *American journal of respiratory and critical care medicine*. NLM (Medline).

[2] Meng L. (2020).

[3] Wilkerson R.G. (2020). Silent hypoxia: a harbinger of clinical deterioration in patients with COVID-19. AJEM (Am. J. Emerg. Med.).

[4] Villarreal-Fernandez E. (2020). ‘A Plea for Avoiding Systematic Intubation in Severely Hypoxemic Patients with COVID-19-Associated Respiratory Failure’, *Critical Care*.

[5] Tobin M.J. (2020). Basing respiratory management of COVID-19 on physiological principles. American Journal of Respiratory and Critical Care Medicine.

[6] MacIntyre C.R., Bui C.M. (2017). Pandemics, Public Health Emergencies and Antimicrobial Resistance - Putting the Threat in an Epidemiologic and Risk Analysis Context.

[7] Truog R.D., Mitchell C., Daley G.Q. (2020). ‘The toughest triage — allocating ventilators in a pandemic’, *New England Journal of Medicine*. Massachussetts Medical Society.

[8] Couzin-Frankel J. (2020). The mystery of the pandemic's ‘happy hypoxia’. Science.

[9] Chandra A., Chakraborty U., Pal J., Karmakar P. (2020 Sep 7). Silent hypoxia: a frequently overlooked clinical entity in patients with COVID-19. BMJ Case Rep..

[10] Gagnier J.J., Kienle G., Altman D.G. (2013). The CARE guidelines: consensus-based clinical case reporting guideline development. Glob Adv Health Med.

